# COUSCOus: improved protein contact prediction using an empirical Bayes covariance estimator

**DOI:** 10.1186/s12859-016-1400-3

**Published:** 2016-12-15

**Authors:** Reda Rawi, Raghvendra Mall, Khalid Kunji, Mohammed El Anbari, Michael Aupetit, Ehsan Ullah, Halima Bensmail

**Affiliations:** 1 Computational Science and Engineering, Qatar Computing Research Institute, Hamad Bin Khalifa University, Doha, Qatar; 2 Division of Biomedical Informatics, Sidra Medical and Research Center, Doha, Qatar

**Keywords:** Residue-residue contact prediction, Shrinkage, GLasso

## Abstract

**Background:**

The post-genomic era with its wealth of sequences gave rise to a broad range of protein residue-residue contact detecting methods. Although various coevolution methods such as PSICOV, DCA and plmDCA provide correct contact predictions, they do not completely overlap. Hence, new approaches and improvements of existing methods are needed to motivate further development and progress in the field. We present a new contact detecting method, COUSCOus, by combining the best shrinkage approach, the empirical Bayes covariance estimator and GLasso.

**Results:**

Using the original PSICOV benchmark dataset, COUSCOus achieves mean accuracies of 0.74, 0.62 and 0.55 for the top *L*/10 predicted long, medium and short range contacts, respectively. In addition, COUSCOus attains mean areas under the precision-recall curves of 0.25, 0.29 and 0.30 for long, medium and short contacts and outperforms PSICOV. We also observed that COUSCOus outperforms PSICOV w.r.t. Matthew’s correlation coefficient criterion on full list of residue contacts. Furthermore, COUSCOus achieves on average 10% more gain in prediction accuracy compared to PSICOV on an independent test set composed of CASP11 protein targets. Finally, we showed that when using a simple random forest meta-classifier, by combining contact detecting techniques and sequence derived features, PSICOV predictions should be replaced by the more accurate COUSCOus predictions.

**Conclusion:**

We conclude that the consideration of superior covariance shrinkage approaches will boost several research fields that apply the GLasso procedure, amongst the presented one of residue-residue contact prediction as well as fields such as gene network reconstruction.

**Electronic supplementary material:**

The online version of this article (doi:10.1186/s12859-016-1400-3) contains supplementary material, which is available to authorized users.

## Background

A multiple sequence alignment (MSA) of orthologous protein sequences not only carries evolutionary sequence information, but also information about functional and structural constraints imposed on the three-dimensional (3D) structure of a protein. Conserved or slightly mutated columns indicate important protein positions for protein stability and function. Additionally, non-conserved positions may also play key roles in maintaining the functionality when accompanied by compensatory mutations at other positions [1, 2]. It is of high interest to develop methods predicting coevolution patterns from MSAs, because coevolving positions mainly involve protein positions proximal in 3D structure [3] and they serve as a valuable source of distance constraints in protein structure [4–7] as well as in protein complex interface predictions [8, 9].

Due to the substantial increase in sequence data in the post-genomic era, a broad range of methods have been introduced for detecting residue-residue contacts from MSAs in the past decades. Mutual information (MI) was one of the first metrics to be applied for contact prediction from MSAs [10, 11]. An improved MI version that corrects for background noise and phylogenetic effects (MIp) has been introduced by Dunn et al. [12]. Recent methodological improvements are able to distinguish between direct and indirect couplings and have demonstrated enormous accuracy in predicting real couplings and coevolution. Such methods include a Bayesian network approach (BvN) [13], Direct Coupling Analysis (DCA) [14, 15], Protein Sparse Inverse COVariance (PSICOV) [16], and pseudolikelihood approaches implemented in plmDCA [17] and GREMLIN [18].

Most recently, new hybrid methods have been developed, amidst many others such as DNCON [19], PConsC [20], CoinDCA [21] or MetaPSICOV [22], where contact detecting methods are combined along with protein physiochemical features to provide more accurate contact predictions.

In the present study, we developed Contact predictiOn Using Shrinked COvariance (COUSCOus), a residue-residue contact detecting method approaching contact inference in a similar manner as PSICOV, by applying the sparse inverse covariance estimation technique introduced by Meinshausen and Bühlmann [23]. Here, we used a different covariance matrix shrinkage approach, the empirical Bayes covariance estimator, which has been shown by Haff to be the best estimator in a Bayesian framework [24], especially dominating estimators of the form *aS*, such as the smoothed covariance estimator applied in PSICOV. By analysing the original PSICOV benchmark test set [16] and proteins from the Critical Assessment of techniques for protein Structure Prediction 11 (CASP11) experiments, we show that COUSCOus significantly outperforms PSICOV. Furthermore, we designed a simple random forest (RF) meta-classifier that includes contact detecting techniques and sequence-derived physiochemical features and showed that replacing PSICOV with COUSCOus enhances the prediction outcome.

## Methods

### Dataset

The benchmark dataset used in this study is the original PSICOV test set introduced by Jones et al. [16]. We used the same alignments without modification as have been made available to ensure comparability. However, for validation we selected 146 out of 150 single domain monomeric proteins because the latest PSICOV version V2.1b3 was unable to provide contact predictions on the remaining four test cases, when used with default parameters. This is due to the insufficient number of effective sequences within the four alignments.

The second test set consists of 37 proteins of the CASP11 experiment (see Additional file 1). We selected only those proteins where the latest version of PSICOV successfully provided predictions, to make a fair comparison. The training set introduced by Jones et al. [22] was used to build the RF meta-classifier.

### Coevolution analysis methods

The residue-residue contact prediction metrics applied in this study are MI [10, 11], MIp [12], OMES [25], BvN [13], DCA [15] and PSICOV [16]. The resulting coevolution between pairs of amino-acids using MI, MIp and OMES were calculated using *Evol* module of Prody [26]. BvN results were generated using Perl scripts and C++ source code kindly provided by the authors [13]. PSICOV results were calculated using the code available online [16]. DCA results were obtained using the fast and free software version FreeContact introduced by Kaján et al. [27]. Methodological details for the different methods may be found in the original studies.

### COUSCOus

#### Pre-processing

In our approach, we generate a sample covariance matrix *S* from the input MSA. The MSAs are composed of *n* orthologous protein sequences where each sequence represents a row. Each protein sequence is made of *m* amino acids as a result of which we have *L* columns per alignment row. The size of the covariance matrix *S* is 21*L*×21*L*. This is because we compute the marginal single site frequencies *f*(*A*_*i*_) and *f*(*B*_*j*_) of observed amino acid types (20 natural occurring amino acids and a gap) in columns *i* and *j* and their corresponding pair site frequencies *f*(*A*_*i*_ *B*_*j*_):

1 $$  S_{ij}^{ab} = f\left(A_{i}B_{j}\right)-f(A_{i})f(B_{j})  $$

Interestingly the precision matrix *Θ* which is the inverse of the covariance matrix *S* will contain the partial correlations of all pairs of variables taking into consideration the effects of all other variables. Hence, the non-zero entries of *Θ* will provide the extent of direct coupling between any two pairs of amino acids at sites *i* and *j*.

Yet, due to the fact that we are generating a covariance matrix *S* out of MSAs representing homologous protein sequences where not all amino acids are present at each site of the MSA, it is certain that *S* is singular and not directly invertible. Several approaches have been proposed to approximate the precision matrix in such cases. The most powerful and widely used technique is the sparse inverse covariance estimation using the graphical lasso (GLasso) [28].

#### GLasso

We briefly summarise the basic motivation and algorithm. Consider matrix *X*=[*X*_1_,…,*X*_*p*_] where *X*_*i*_ is a random vector of length *n* with covariance matrix *Σ* and precision matrix *Θ*={*θ*_*ij*_}_1≤*i*,*j*≤*p*_. Further, let *S* denote the empirical covariance matrix obtained from the data. The estimation of the precision matrix *Θ* is challenging when it is sparse. Interestingly, this task is closely related to selection of graphical models.

Let *G*=(*V*,*E*) be a graph representing conditional independence relations between components of *X*. *G* is composed of a set of vertices *V* with *p* components {*X*_1_,…,*X*_*p*_} and an edge set *E* of ordered pairs (*i*,*j*), with (*i*,*j*)∈*E*, if an edge between *X*_*i*_ and *X*_*j*_ exists. The edge between *X*_*i*_ and *X*_*j*_ is excluded from the edge set *E* if and only if *X*_*i*_ and *X*_*j*_ are independent given all other components {*X*_*k*_,*k*≠*i*,*j*}. Assuming that the raw data X is multivariate gaussian (*X*∼*N*(*μ*,*Σ*)), the conditional independence between *X*_*i*_ and *X*_*j*_ given all other components is equivalent to zero in the precision matrix (*θ*_*ij*_=0) as shown in [29]. Hence, for gaussian distributions recovering the structure of graph *G* is equivalent to the estimation of the support of the precision matrix.

The precision matrix *Θ* can then be estimated using a *L*_1_ penalised log-likelihood approach. The GLasso algorithm, introduced by Friedman et al. [28], efficiently computes the solution by:

2 $$  \hat{\Theta}_{\text{GLasso}} := \arg\min_{\Theta \succ 0}\left\{\text{tr}(S\Theta) - \log\det(\Theta) + \lambda\|\Theta\|_{1}\right\},  $$

with *tr* as trace, ∥*Θ*∥_1_ as the sum of the absolute values of the elements in *Θ* and *λ* as a positive tuning parameter to control the sparsity.

#### Empirical Bayes covariance estimator

The most natural estimator of the covariance *Σ* is the sample covariance matrix *S*. The estimator is optimal in the classical settings with large number of samples and fixed low dimensions (*n*>*p*). However, it performs poorly in high-dimensional settings (*n*<<*p*), see Johnstone [30]. The GLasso approach operates very well in this context, but the computational time required to reach convergence can be large in some cases such as for protein families with low number of sequences. As an alternative to the natural estimator *S*, several shrinkage estimators have been proposed in the literature [31, 32]. They take a weighted average of the sample covariance matrix *S*, with a suitable chosen target diagonal matrix. Jones et al. applied a smoothed covariance estimator that shrinks the matrix towards the shrinkage target $F=diag(\bar {S},\bar {S},\ldots,\bar {S})$ [16]. In this work, we applied the empirical Bayes estimator proposed by Haff [24]:

3 $$  \hat{\Sigma}=S+\frac{p-1}{n\ tr(S)}I_{p},  $$

where *I*_*p*_ represents the identity matrix of order *p*. In a Bayesian framework, it has been proven by Haff that this estimator is the best estimator of the form *a*(*S*+*u* *t*(*u*)*C*), with 0<*a*<1/(*n*−1) and *u*=1/tr(*S* ^−1^ *C*). Here *t*(·) is non-increasing and *C* an arbitrary positive definite matrix. It dominates estimators of the form *aS* by a substantial amount. More precisely, it has been proven that under the *L*_2_ loss, the uniform reduction in the risk function is at least $100\frac {p+1}{n+p}$%. In this study, we performed the shrinkage until the adjusted covariance matrix $\hat {\Sigma }$ is no longer singular, i.e. is a positive-definite matrix. The adjusted covariance matrix $\hat {\Sigma }$ was finally applied in the GLasso algorithm to obtain the sparse precision matrix, that contains the degree of direct coupling between any pair of amino acids.

#### APC correction

The coevolution pair list is generated identically to the PSICOV final processing. For each MSA column pair *i* and *j* we compute the *L*_1_-norm out of the corresponding 20×20 submatrix in *Θ* (only contributions of the 20 amino acid types are considered):

4 $$  C_{ij} = \sum_{ab}\mid \Theta_{ij}^{ab}\mid  $$

Furthermore, we adjust the coupling score by the average product correction (APC), previously applied for MI by Dunn et al. [12] to reduce entropic and phylogenetic bias:

5 $$  {COUSCOus}_{ij} = C_{ij} - \frac{\bar{C}_{(i-)}\bar{C}_{(-j)}}{\bar{C}}  $$

with $\bar {C}_{(i-)}$ as the mean precision norm of column *i* and all other columns, $\bar {C}_{(-j)}$ as the corresponding for column *j* and $\bar {C}$ as the mean precision norm of all coupling scores.

### Random forest meta-classifier

As previously indicated, new hybrid methods that combine coevolution detecting tools with other sources of information such as protein physiochemical features outperforms single methods like PSICOV. However, we are convinced that improvements of single residue-residue contact detecting methods can boost new emerging hybrid techniques. We designed a RF meta-classifier that includes several contact prediction methodologies along with a small number of sequence-derived physiochemical features.

In particular, we built a RF classifier using the training set alignments from the MetaPSICOV study [22]. In total, we used 336 protein alignments where PSICOV was able to successfully provide contact predictions. The RF was trained using the following features:

- Contact detecting methodologies MI, MIp, BvN, PSICOV or COUSCOus, FreeContact and CCMpred
- Secondary structure and solvent exposure probabilities derived from PSIPRED [33]
- Shannon entropy using R [34] package bio3d [35]
- Hydrophobicity using R package Interpol [36]
- Amino acid physiochemical properties

The RF meta-classifier was trained using 500 trees with ten features and a max-depth of eight. We performed five-fold cross-validation while training the classifier and optimised the area under the curve (AUC) metric for performance.

### Evaluation metrics and distance descriptions

The problem of predicting protein residue-residue contacts is well-known to be an extremely difficult one as on average only 3% of all possible residue pairs in known protein structures are identified to be real contacts. In the latest CASP11 challenge [37], this problem was tackled by dividing the contact prediction task into two categories. First, evaluation of predicted contacts using quality metrics like Accuracy and *X*_*d*_ [38] on reduced lists (RL). Second, evaluation of predicted contacts using quality metrics like Matthew’s correlation coefficient (*MCC*) [39] and area under the precision-recall (*A* *U* *C*_*pr*_) for full lists (FL). The RL are usually defined by considering the top *L*/*n* predicted contacts where *L* is the length of the evaluation target or protein sequence and *n* is a small integer (e.g. 1,5 or 10). RL metric accuracy is calculated as $\frac {TP}{TP+FP}$ where TP defines a correctly predicted contact and *FP* an incorrectly predicted contact. The second RL metric *X*_*d*_ represents the difference between the distance distributions of the predicted contacts and all pairs distance distributions in the 3D target structure. It is defined as $X_{d} = \sum \limits _{i=1}^{15} \frac {Pip-Pia}{di*15}$, with *Pia* and *Pip* are the percentages of pairs included in the *i* ^*th*^ bin for the whole target and predicted contacts respectively. Additional details can be found in [38]. The FL metrics used in this study are *A* *U* *C*_*pr*_ as it is a robust metric for unbalanced classes and the Matthew’s correlation coefficient [39] to evaluate all residue pairs for contact prediction.

## Results and discussion

**Fig. 1:**
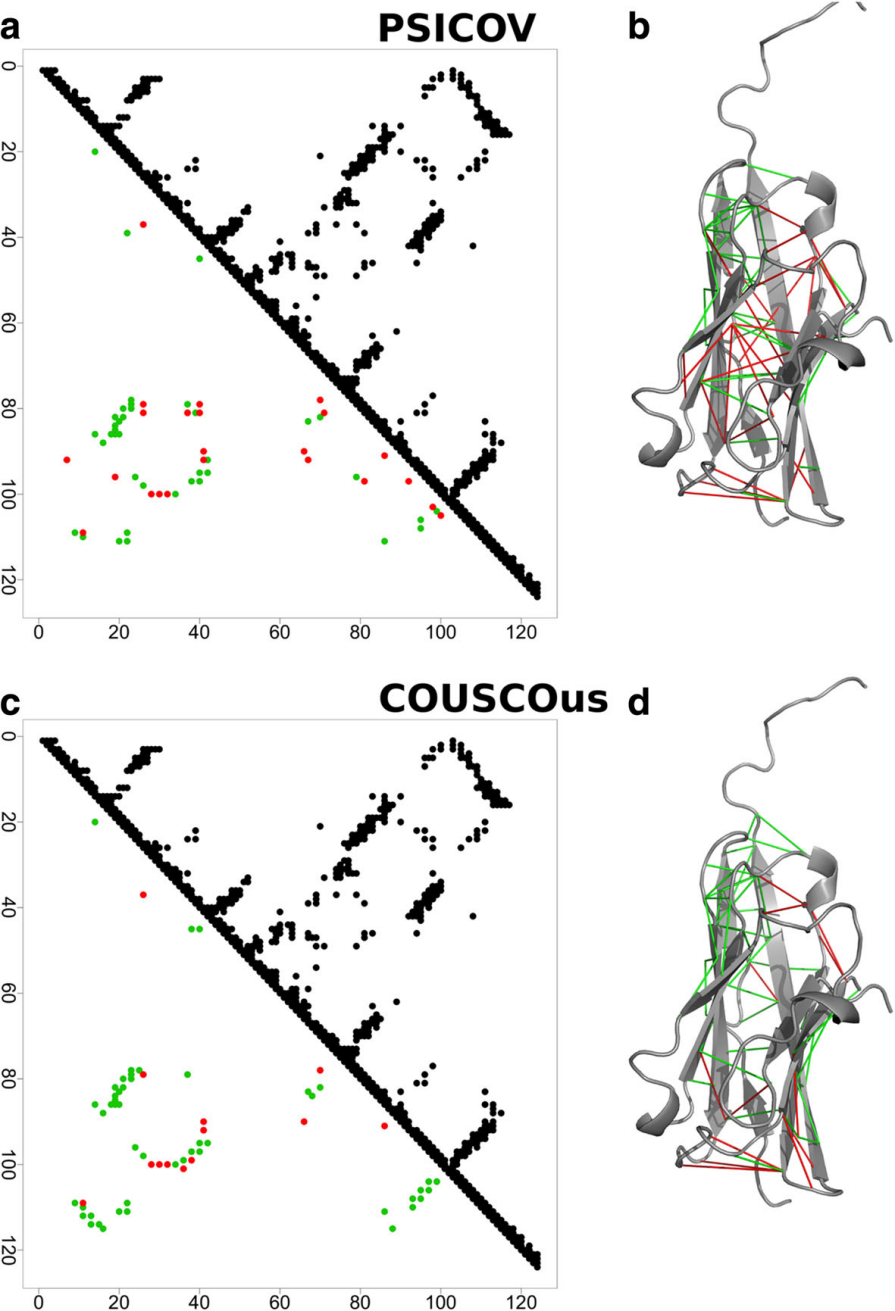
The *top* *L*/2 (in this case 62) *long*, *medium* and *short* contact predictions for the Immunoglobulin V-set domain family (PFAM ID: PF07686) obtained using PSICOV (**a** and **b**) and COUSCOus (**c** and **d**) and mapped to the myelin oligodendrocyte glycoprotein 3D crystal structure (PDB ID: 1PKO) (*right panel*). Correctly predicted contacts are shown in *green* and incorrect ones in *red*. *Upper triangles* of the contact maps display all the native *C*_*β*_−*C*_*β*_ contacts (*left panel*). The *lower triangles* show contacts predicted by PSICOV (**a** and **b**) and COUSCOus (**c** and **d**)

We first illustrate as an example in Fig. 1 the spatial proximity of the predicted contacts obtained by PSICOV (a and b) and COUSCOus (c and d) for the Immunoglobulin V-set domain (Protein family database [40] (PFAM) ID: PF07686). The upper triangles of the presented contact maps (Fig. 1 a and c) display the native contacts. A residue-residue pair is hereby considered to be in contact if the two amino acids are proximal in the 3D structure, in particular if their *C*_*β*_- *C*_*β*_ (*C*_*α*_ in the case of glycine) distance is less than 8 Å ngström (Å). The lower triangles show the *L*/2 contact predictions (in this case 62) obtained by using either PSICOV or COUSCOus. Correctly predicted contacts are coloured in green and incorrect ones in red. Further, we mapped the top *L*/2 predictions to the structure of the myelin oligodendrocyte glycoprotein (Protein Data Bank [41] (PDB) ID: 1PKO), solved at 1.45 Å resolution (Fig. 1 b and d). Out of 62 possible contacts, COUSCOus correctly predicted 49 (accuracy: 0.79) compared to 39 (accuracy: 0.63) by PSICOV resulting in higher accuracy of COUSCOus. The figure shows (b) that the incorrect identified pairs are mainly located in loop regions at the top and the bottom. Hence, the pairs may still have distances less than 8Å considering that the unordered regions are not static as illustrated by a crystal structure. In contrast, incorrect predicted pairs from PSICOV (Fig. 1 d), are distributed over the entire protein.

The performance of COUSCOus was evaluated on the original PSICOV benchmark test set using standard assessment metrics applied in CASP: accuracy, *X*_*d*_,*M* *C* *C* and *A* *U* *C*_*pr*_ (see Methods). We distinguished between three types of contacts: short (6 ≤ residue separation < 12), medium (12 ≤ residue separation < 24) and long range contacts (residue separation ≥ 24).

We list in Table 1 the mean accuracies of COUSCOus and PSICOV for the top- *L*/10, top- *L*/5 and top-*L* for long, medium and short range predicted contacts on the original PSICOV benchmark set. For the top- *L*/10 predictions COUSCOus is 10, 8 and 13% more accurate than PSICOV for long, medium and short range contacts. Similarly, for the top- *L*/5 predictions we observed the similar gains in accuracy when using COUSCOus. For the top-*L* predictions we observed different accuracies for the three types of contacts. The gain in accuracy of 11% when using COUSCOus was similar for the long range contacts but the gain dropped to 6 and 5% for medium and short range contacts.

**Table 1:** Mean accuracies of COUSCOus vs. PSICOV on PSICOV benchmark dataset

	Accuracy top- *L*/10			Accuracy top- *L*/5			Accuracy top-*L*		
	Long	Medium	Short	Long	Medium	Short	Long	Medium	Short
PSICOV	0.6724	0.5709	0.4876	0.5816	0.4401	0.3716	0.3016	0.1787	0.1589
COUSCOus	**0.7394**	**0.6151**	**0.5509**	**0.6494**	**0.4837**	**0.4037**	**0.3341**	**0.1892**	**0.1664**

Higher mean accuracies in bold

The second evaluation metric applied in this study, the *X*_*d*_ score, estimates the deviation of the distribution of distance in the RL sets (*L*/10,*L*/5 or *L*) of contacts from the distribution of the distances in all residue pairs within the protein (see Methods). Table 2 summarises the average *X*_*d*_ scores for COUSCOus and PSICOV. For the top-*L* predictions COUSCOus is more accurate than PSICOV on long, medium and short range contacts (16, 10 and 18%). For the top- *L*/10 and top- *L*/5 predictions we observed smaller improvements in *X*_*d*_ score ranging from 1 to 11%. Moreover, we compared the performance of COUSCOus and PSICOV on FL; considering all possible residue pairs. In Table 3 we summarise the mean *A* *U* *C*_*pr*_ values of the precision recall (PR) curves for COUSCOus and PSICOV. COUSCOus outperforms PSICOV with gains in accuracy of 14, 11 and 11% for long, medium and short range contacts, respectively.

**Table 2:** Mean *X*_*d*_ values of COUSCOus and PSICOV on PSICOV benchmark dataset

	*X*_*d*_ top- *L*/10			*X*_*d*_ top- *L*/5			*X*_*d*_ top-*L*		
	Long	Medium	Short	Long	Medium	Short	Long	Medium	Short
PSICOV	0.2694	0.2751	0.2239	0.2518	0.2564	0.2068	0.1930	0.1864	0.1422
COUSCOus	**0.2816**	**0.2788**	**0.2302**	**0.2718**	**0.2646**	**0.2295**	**0.2239**	**0.2058**	**0.1671**

Higher mean values in bold

**Table 3:** Mean AUC _*pr*_ values

	Long	Medium	Short
PSICOV	0.2150	0.2630	0.2715
COUSCOus	**0.2447**	**0.2930**	**0.3014**

Higher mean values in bold

**Fig. 2:**
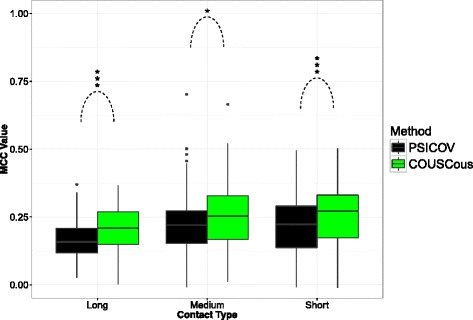
MCC distributions for PSICOV benchmark proteins in case of long, medium and short range contacts predicted by PSICOV and COUSCOus. The stars represent statistical significance where ⋆ is used to represent *P*-value < 0.05 and ⋆⋆⋆ is used to represent *P*-values < 0.001

We also performed an exhaustive analysis of COUSCOus and PSICOV w.r.t. *MCC* for long, medium and short range contact predictions. In Figure 2 we illustrate via box plot the distribution of the *MCC* values for the two prediction methods. It is apparent from the box plots that COUSCOus is superior in predicting real residue contacts. To further test for significance we performed a t-test, after successfully testing for normal distribution and variance homogeneity, on the *MCC* distributions for different contact ranges. COUSCOus outperforms PSICOV on all types of contacts significantly, with *P*-values of 6×10^−7^,1.2×10^−2^ and 6×10^−4^ for long, medium and short range contacts, respectively.

**Fig. 3:**
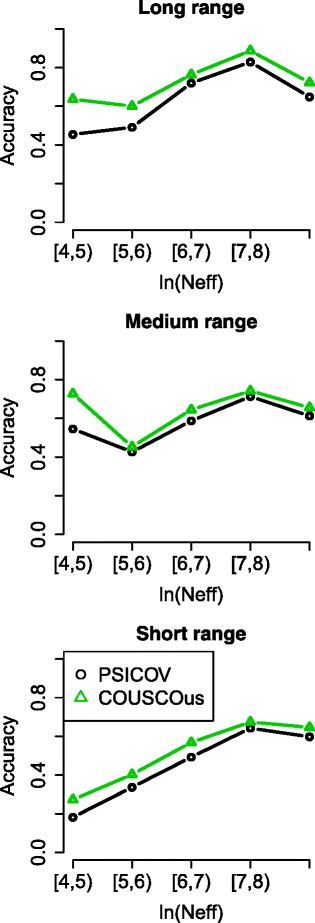
Dependence of the performance of PSICOV and COUSCOus on the effective number of sequences (*N*_*eff*_) in the MSAs. The performance is evaluated using accuracies for the *top* *L*/10*long*, *medium* and *short* contacts. The *solid line* represents the averaged accuracies of the test set binned into five different categories of Neff (ln(Neff): [4, 5), [5, 6), [6, 7), [7, 8), [8, 10)). COUSCOus outperforms PSICOV independent of the *l* *n*(*N*_*eff*_) in the test set

Next, we analysed the dependence of the performance of PSICOV and COUSCOus with regard to the size of the protein family. In this case, we used the number of effective sequences in a MSA as comparison metric to account for the fact that highly similar homologous do not provide any additional contact information than a single one. Similar to Ma et al. [21] we grouped the test set members into five categories by *l* *n*(*N*_*eff*_): [4,5),[5,6),[6,7),[7,8),[8,10), and calculated the averaged *L*/10 accuracies for each group. Figure 3 shows clearly that COUSCOus outperforms PSICOV regardless of the *l* *n*(*N*_*eff*_) on long, medium and short range contacts. In addition, we tested the performance of COUSCOus and PSICOV on an independent test set from the latest CASP11 experiment. In Table 4 we show the mean accuracies of COUSCOus and PSICOV for the top- *L*/10, top- *L*/5 and top-*L* for long, medium and short range predicted contacts. For the top- *L*/10 predictions COUSCOus is 10, 9 and 13% more accurate than PSICOV for long, medium and short range contacts, respectively. Similarly, for the top- *L*/5 predictions we observed gains in accuracy of 13, 10 and 9% when using COUSCOus. For the top-*L* predictions we observed different accuracies for the three types of contacts. A gain in accuracy of 13% is observable when using COUSCOus for the long range contacts but the gain dropped to 4 and 8% for medium and short range contacts.

**Table 4:** Mean accuracies of COUSCOus vs. PSICOV on the CASP11 benchmark dataset

	Accuracy top- *L*/10			Accuracy top- *L*/5			Accuracy top-*L*		
	Long	Medium	Short	Long	Medium	Short	Long	Medium	Short
PSICOV	0.6687	0.5809	0.5278	0.5872	0.4809	0.4229	0.3383	0.2373	0.1820
COUSCOus	**0.7385**	**0.6335**	**0.5965**	**0.6610**	**0.5285**	**0.4636**	**0.3828**	**0.2477**	**0.1958**

Higher mean accuracies in bold

Next, we designed two experiments using a RF meta-classifier where we combine contact detecting tools with protein sequence derived features. In the first case we used predictions of PSICOV as a feature in the meta-classifier and in the second case we replaced PSICOV predictions with COUSCOus predictions. The classifier including COUSCOus results as a feature outperforms the classifier including PSICOV results for the top- *L*/10 and top- *L*/5 predictions on all types of contacts except for the top-*L* predictions for long range contacts (see Table 5).

**Table 5:** Mean accuracies of RF meta-classifier including COUSCOus or PSICOV as a feature on the PSICOV benchmark dataset

	Accuracy top- *L*/10			Accuracy top- *L*/5			Accuracy top-*L*		
	Long	Medium	Short	Long	Medium	Short	Long	Medium	Short
RF-PSICOV	0.7846	0.6946	0.6547	0.7047	0.5500	0.5140	**0.3991**	0.2439	0.2212
RF-COUSCOus	**0.7881**	**0.7065**	**0.6618**	**0.7112**	**0.5674**	**0.5191**	0.3984	**0.2453**	**0.2225**

Higher mean accuracies in bold

In Additional file 2 we illustrate the mean accuracies of different contact detecting techniques. Our newly developed technique COUSCOus (green upper triangle) outperforms PSICOV (black points) and is equally well as FreeContact (red lower triangle) on all contact types. The best single contact detecting tool is CCMpred (magenta rectangle). Our simple RF meta-classifier that combines 6 single residue-residue contact detecting tools along with 4 sequence-derived features outperforms all single methods. However, MetaPSICOV, a multi-stage neural network hybrid method that combines five coevolution techniques along with a broad range of sequence-derived features is still the best performing method.

## Discussion

In this work, we assessed the performance of our newly developed method COUSCOus in predicting residue-residue contacts from MSAs. In particular, the performance was tested in comparison to PSICOV on the original PSICOV benchmark test set as well as on CASP11 targets. On the RL sizes COUSCOus outperformed PSICOV substantially, with on average 10% gain in prediction accuracy for all types of contacts. Moreover, COUSCOus proved to be superior over PSICOV on FL sizes with average *A* *U* *C*_*pr*_ gains of 12%. With regard to *MCC* scores COUSCOus is even significantly outperforming PSICOV, illustrated with the help of box plots and hypothesis tests (see also Additional file 3). Further, we reported that COUSCOus’s gain in accuracy is independent of the number of effective sequences in a given MSA.

The main motivation of this work was to highlight that improvements of single residue-residue contact detecting tools, in this case PSICOV, might lead to improvements of new hybrid methods that combine contact detecting techniques with physiochemical and other sequence derived protein features. As proof of concept, we showed with the help of a simple RF meta-classifier that PSICOV should be replaced in hybrid classifiers by COUSCOus.

## Conclusion

Jones et al. [16] demonstrated in their initial work that GLasso in principle performs excellently in identifying directly coupled columns within a MSA. In the present study, we highlighted that the application of a different shrinkage approach than the one used in PSICOV, the empirical Bayes covariance estimator, in combination with GLasso substantially increased the contact precision. The theoretically shown superiority of the empirical Bayes covariance estimator over simpler smoothed covariance estimators of the form *aS* is also valid within this application of contact detection from MSAs.

Furthermore, it is worth mentioning that other research fields that apply the GLasso procedure, such as gene network reconstruction, may also benefit from applying other shrinkage techniques. We are keen to investigate the effect of shrinkage in other graphical inference problems in future work.

Another important application that we are keen to investigate in future is the de novo structure prediction of proteins or protein complexes using COUSCOus or a hybrid classifier, including COUSCOus contact predictions as distance constraints, similarly to what have been applied in EVFold [4] and EVComplex [8], or PconsFold [42].

## Additional files

Additional file 1 A complete list of all CASP11 protein codes applied in this study. (PDF 72.9 kb)

Additional file 2 Mean accuracies of several contact detecting methods for the top- *L*/10,*L*/5 and *L* predictions for all contact types applying the PSICOV benchmark dataset. (PDF 85.6 kb)

Additional file 3 Scatterplot comparing the accuracies of the top L contacts of PSICOV to COUSCOus, using sequence separation ≥6. (PDF 78 kb)

## Abbreviations

3D: three-dimensional
Å: Å ngström
AUC: area under the curve
BvN: Bayesian network approach
COUSCOus: Contact predictiOn Using Shrinked COvariance
CASP11: Critical Assessment of techniques for protein Structure Prediction 11
DCA: Direct Coupling Analysis
FL: full lists
GLasso: graphical lasso
MCC: Matthew?s correlation coefficient
MI: mutual information
MIp: corrected mutual information
MSA: multiple sequence alignment
PDB: Protein Data Bank
PFAM: Protein family database
PR: precision recall
PSICOV: Protein Sparse Inverse COVariance
RF: Random forest
RL: reduced lists
