# Supplementary material for: COUSCOus: improved protein contact prediction using an empirical Bayes covariance estimator
Source: BMC Bioinformatics. 2016 Dec 15;17:533. doi: 10.1186/s12859-016-1400-3 (PMC5159955; doi:10.1186/s12859-016-1400-3)
Supplement: Additional file 2 — Mean accuracies of several contact detecting methods for the top- L/10,L/5 and L predictions for all contact types applying the PSICOV benchmark dataset. (PDF 85.6 kb) [file 12859_2016_1400_MOESM2_ESM.pdf]

Additional file 2 for:

COUSCOus: Improved protein contact prediction using an empirical Bayes covariance estimator

Reda Rawi [1,*], Raghvendra Mall [1], Khalid Kunji [1], Mohammed El Anbari [2], Michael Aupetit [1], Ehsan Ullah [1] and Halima Bensmail [1]

[1]Computational Science and Engineering, Qatar Computing Research Institute, Hamad Bin Khalifa University, Doha, Qatar and
[2]Division of Biomedical Informatics, Sidra Medical and Research Center, Doha, Qatar.

∗ Corresponding author
E-mail: rrawi@qf.org.qa

List of CASP11 proteins applied in this study (Target IDs).

T0762	T0764	T0765	T0768	T0772
T0774	T0776	T0778	T0780	T0782
T0787	T0788	T0792	T0798	T0800
T0801	T0805	T0807	T0811	T0812
T0813	T0815	T0817	T0819	T0821
T0823	T0824	T0825	T0829	T0841
T0843	T0845	T0847	T0849	T0851
T0854	T0856			
