# Supplementary material for: COUSCOus: improved protein contact prediction using an empirical Bayes covariance estimator
Source: BMC Bioinformatics. 2016 Dec 15;17:533. doi: 10.1186/s12859-016-1400-3 (PMC5159955; doi:10.1186/s12859-016-1400-3)
Supplement: Additional file 3 — Scatterplot comparing the accuracies of the top L contacts of PSICOV to COUSCOus, using sequence separation ≥6. (PDF 78 kb) [file 12859_2016_1400_MOESM3_ESM.pdf]

Additional file 1 for:

COUSCOus: Improved protein contact prediction using an empirical Bayes covariance estimator

Reda Rawi[1,*], Raghvendra Mall [1], Khalid Kunji [1], Mohammed El Anbari [2], Michael Aupetit [1], Ehsan Ullah [1] and Halima Bensmail[1]

[1]Computational Science and Engineering, Qatar Computing Research Institute, Hamad Bin Khalifa University, Doha, Qatar and
[2]Division of Biomedical Informatics, Sidra Medical and Research Center, Doha, Qatar.

$*$ Corresponding author
E-mail: rrawi@qf.org.qa

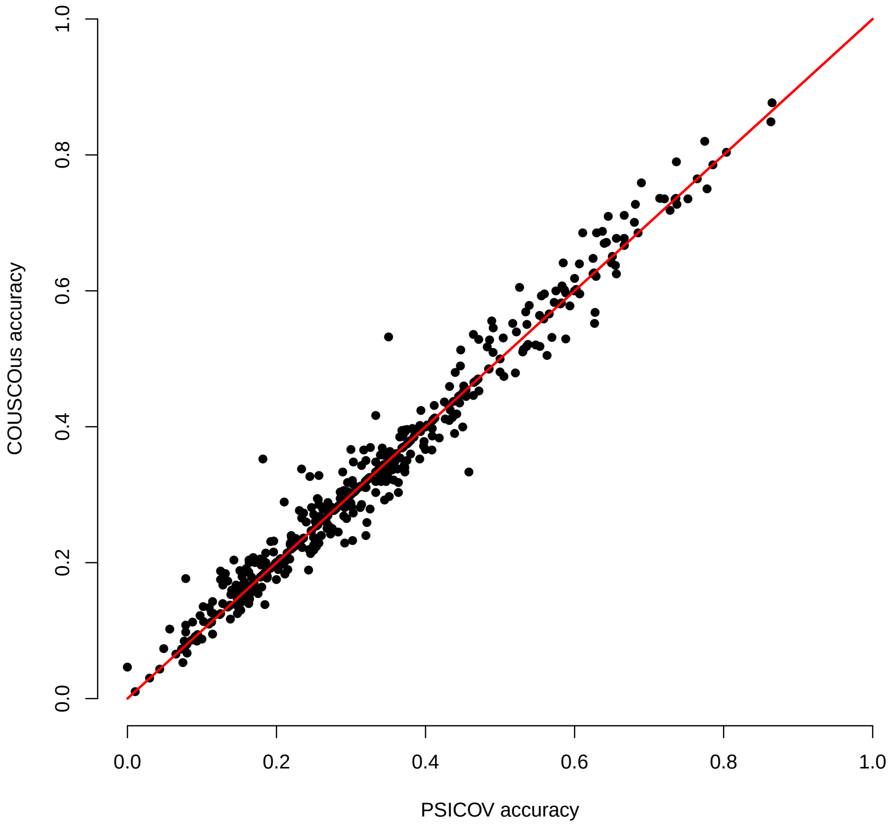

Figure S2: Scatterplot comparing the accuracies of the top L contacts of PSICOV to COUSCOus, using sequence separation ≥ 6.
